# Engaging patients and public in decision‐making: approaches to achieving this in a complex environment

**DOI:** 10.1111/hex.12550

**Published:** 2017-03-16

**Authors:** Mary Chambers

**Affiliations:** ^1^ Faculty of HealthSocial Care and EducationKingston University and St. George’s University of London

We are living in a dynamic, rapidly changing world with a more informed society, members of which hold greater expectations of health‐care services and treatment outcomes. Consequently, the nature and delivery of health care is constantly changing, influenced by advances in medical science and technology, demography and greater public awareness of health and illness. These transformations have bearing on the dynamics of relationships that exist between patients and health‐care professionals. A key change is the balance of power within relationships with a move away from the previous paternalistic approach, to one of partnership working and shared decision‐making (SDM) based on currently available evidence.^1^

If care is to be person‐centred, then patients must be at the centre of decision‐making as outlined in the Department of Health Liberating the NHS – no decision about me without me.^2^ For this paradigm shift to be successful, education and support for both patients and professionals is essential. Patients require information and knowledge to meaningfully contribute to decision‐making; professionals require the skills and confidence to give patients “permission” to have their say and accept that they too are experts when it comes to their health and illness. For both groups, there are a range of tools available to support the decision‐making process, but these are not universally accepted.

This issue of *Health Expectations* includes a range of papers exploring different aspects of patient participation in SDM, and the wider contribution of patient and public to a variety of care initiatives and service evaluations. In keeping with the philosophy of HEX, the papers offer an international perspective, utilize a variety of research methodologies and highlight some of the key challenges surrounding change for both patients and professionals.

Patients require up‐to‐date evidence‐based information to enable them to take informed care decisions. *Prothero et al*. report how a handbook for patients with moderate rheumatoid arthritis can support intensive management. The benefits of providing patients with information are acknowledged as increasing patient's knowledge, satisfaction and adherence to treatment. In developing patient information material, it is essential that the material is codesigned^3^, ^4^ with patients and/or carers to ensure it meets quality standards, is relevant and easy to understand in order to increase the likelihood of it being helpful and used. Previously co‐design was a neglected area, with information being prepared, largely based on a biomedical model, by professionals and given to patients rather than enhancing patient autonomy.

To embrace the essence of patient‐centred care^5^ and SDM, the imparting and exchange of information and knowledge are essential. This can be challenging for professionals as demonstrated in the paper by *Lazenby et al.,* in the context of end of life care and *Engelen et al*. with respect to exploring the views of men and general practitioners (GPs) on using decision aids for the early detection of prostate cancer.

There are commonalities in the findings of these two studies concerning the sharing of information and decision‐making. For example, with respect to end of life care, prognosis regarding end‐stage renal disease was reported to be rarely discussed with patients unless specifically asked for, professionals being of the view that patients do not want, or need to know, this information. This highlights the challenges faced by practitioners when having to engage in difficult conversations; similarly, in the study of the early diagnosis prostate cancer, the men were of the opinion that they should all be tested and suggested the need for a short decision‐aid for use during consultations. General practitioners reported many disadvantages of early diagnosis and that decision tools were too time‐consuming. The conflicting views of men and GPs led to different expectations or goals: men expected their GPs to test them proactively; GPs reported being aware of these expectations, but avoided bringing up early diagnosis during the consultation to avoid suggesting that testing was available. Some GPs felt thwarted in their efforts to keep silent by other members of the care team who advised men to get tested. These differing views led to a clash of expectations, fracturing of relationships and reduced opportunity for SDM with the potential for interpersonal conflict in the team.

Currently, all care and treatment is conducted within the context of teams, which are usually multidisciplinary and considered necessary for effective patient‐centred care. Membership of care teams is generally based on the understanding of professionals without consideration of the patients’ perspectives. Given that patients are the focus of team endeavours, it seems appropriate to consider their perspective. A Canadian study conducted by *LaDonna et al*. focused on patients with heart failure to see how they conceptualized their care team and perceived their own and other team members’ roles. The study highlighted a broader conceptualization of team membership as perceived by patients based largely on level of contact. Patients regarded themselves as active members of the team. This would suggest that in future those with chronic conditions should be invited to share who they consider are the members of their care team and how they perceive their own role within the team. Additionally, practitioners could consider what the team looks like from the patient's perspective.

For self‐care to be successful, it needs be located in a supportive environment both at practitioner level and at organizational level. The study by *Morgan et al*. could be considered as setting the scene at practitioner level by synthesizing research into professional practitioners’ perspectives, practices and experiences to inform a reconceptualization of support for self‐management. From the synthesis, two categories of support emerged, one narrow and the other broad. The narrow view is where practitioners practice within the parameters of biomedical indicators and are disease‐focused, with no emphasis on other aspects of patients’ lives. The broader conceptualization of support puts emphasis on partnership working based on trust and being “present” for the patient and promotes problem‐solving. At the organizational level, *Nickel et al*. report on a study looking at organizational change at different levels and the relationship between institutions and self‐help groups. Self‐help friendliness (SHF) was developed as an approach to implement wider co‐operation between self‐help associations and health‐care services. The goal of SHF is to involve patients as much as possible, to avoid an over‐reliance on the perspective of health professionals and include patients in the quality management of health‐care institutions. There are marked similarities between the broad view of self‐help support as outlined by *Morgan et al.,* and the values underpinning SHF, for example partnership working, patient autonomy and well‐being. The conclusion to be drawn is that for self‐help to flourish there should be synergy between the organizational culture (SHF) and the attitudes and behaviour of professionals.

The media can play an active role in raising awareness of health‐care issues either positively or negatively. The study by *Hanson et al*. aimed to explore how newspaper articles present stories about medical research and how people interpret and use them. They concluded that newspaper articles relating to research into new drugs and medical technologies were positive and that patients and carers read stories about medical research critically and sometimes with cynicism. Despite this scepticism, the patient participants in the studies reported by *Lazenby et al*. and *Engelen et al*. suggested that the media could/should be engaged in raising awareness about conditions and interventions.

Obtaining and measuring outcomes in health and social care are important for a number of reasons. For example, how do patients experience their care and treatment? How easy is it for patients to access services? Are services appropriate to meet patient and community needs? What are the outcomes of clinical consultations? Are services delivering value for money? To answer these and other related questions, it is important to have reliable and valid instruments that are easy to use and result in meaningful data of value to clinicians, health service providers, commissioners and patients. This issue of HEX highlights examples of different approaches being developed to do just that *Haggerty et al*. report a measure of availability and accommodation of health care that is valid for rural and urban contexts, and predicts consequences of difficult access for patient‐initiated care. Accessing a service, especially if the first contact has the potential to encourage (or deter) future engagement, is therefore highly important to the patient experience.

Although initially being developed and tested in a different health‐care setting, the *Haggerty et al*. study interfaces with that of *Murphy et al*. who describe the development of a Patient Reported Outcome Measure for primary care.

Engaging the public in consultation around policy decisions is deemed to aid transparency of decision‐making about issues that impact on communities, not infrequently such consultations are met with scepticism. The study by *Campbell et al. i*nvestigated the changes made to draft guidance on interventional procedures for the UK National Health Service, following public consultation. This study suggests that public consultation can result in changes being made to guidance. It also offers reassurance about the authenticity and credibility of the process as well as helping to silence the critics.

Previous issues of HEX have looked at the contribution of patients to the research process but not many (if any) addressed the question of why some patients want to engage in research. Using Q‐Methodology, *Meshaka et al*. attempted to answer this question. The study population were healthy pregnant woman but at risk of diabetes. The authors suggest that the reasons for engagement in research were an interest in helping the advancement of medical research, a personal connection to the disease and lack of inconvenience as the data were collected during a routine clinic visit. We suspect that the same reasons for engagement may apply to other patient groups; if readers of HEX are engaged in this area of research, then consider submitting a manuscript to enable on‐going discussion.
